# Effective Treatment of Primary Orbital Squamous Cell Carcinoma with Radiotherapy: A Rare Case Report and Literature Review Update

**DOI:** 10.1007/s13193-024-02096-5

**Published:** 2024-09-20

**Authors:** Jyotiman Nath, Abhinandan Das, Debasmita Saha, Shiraj Ahmed, Kaberi Kakati, Anamika Dutta

**Affiliations:** 1 Department of Radiation Oncology, Dr B Borooah Cancer Institute, Guwahati, Assam, 781016 India; 2 Department of Oncopathology, Dr B Borooah Cancer Institute, Guwahati, India; 3 Department of Head and Neck Surgery, Dr B Borooah Cancer Institute, Guwahati, India

**Keywords:** Orbit, Radiotherapy, Squamous cell carcinoma

## Abstract

Primary squamous cell carcinoma (SCC) of the orbit is exceptionally rare, comprising only a small fraction of ocular tumours, with most cases being secondary to tumours originating from nearby structures or due to distant metastasis. We present a case of primary orbital SCC in a 60-year-old male, discussing its diagnostic workup, imaging findings, and multidisciplinary management. The patient underwent definitive radiotherapy with concurrent chemotherapy after being deemed inoperable. Without any severe treatment-related adverse event, the patient achieved a complete metabolic response at 7 months post-treatment with maintained vision. As evident from a review of the literature, treatment approaches vary widely in this group of patients, with radiotherapy emerging as a successful modality in achieving disease control while preserving vision with acceptable toxicity. This case underscores the complexity of managing primary orbital SCCs and highlights the efficacy of radiotherapy in achieving favourable outcomes.

## Introduction

6.8% of histologically confirmed ocular tumours are secondary squamous cell carcinomas (SCCs). These most frequently originate from the paranasal sinuses, followed by the nasal cavity, nasopharynx, periocular skin, epibulbar structures, and lacrimal sac [1].

Due to the lack of native squamous epithelium in the orbit, primary squamous cell carcinoma of the orbit is a very rare condition. Till date, only a handful of such cases have been reported in the literature. The characteristic features, which include orbital proptosis, diplopia, restriction of eye movement, and compressive neuropathy, are similar to other orbital lesions.

Owing to the rarity of the condition, there is no consensus regarding management of the same. We discuss the workup, imaging, multidisciplinary discussion-based decision, and treatment of a case of primary orbital SCC in our institute.

## Case Summary

A 60-year-old male with a known history of hypertension experienced redness, blurred vision, and severe pain radiating to his head. He underwent cataract surgery at a local hospital, but symptoms persisted, leading him to a higher centre.

**Fig. 1:**
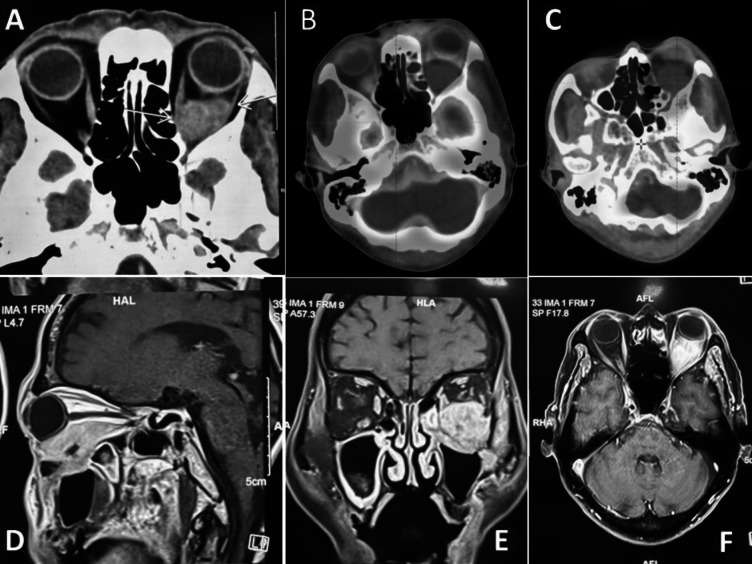
Pre-treatment images. **a** Axial CT; **b**, **c** axial PET-CT; **d** CEMRI sagittal, **e** CEMRI coronal, and **f** CEMRI axial showing an irregular mass lesion in the left orbit in retroorbital space predominately in the inferior part

**Fig. 2:**
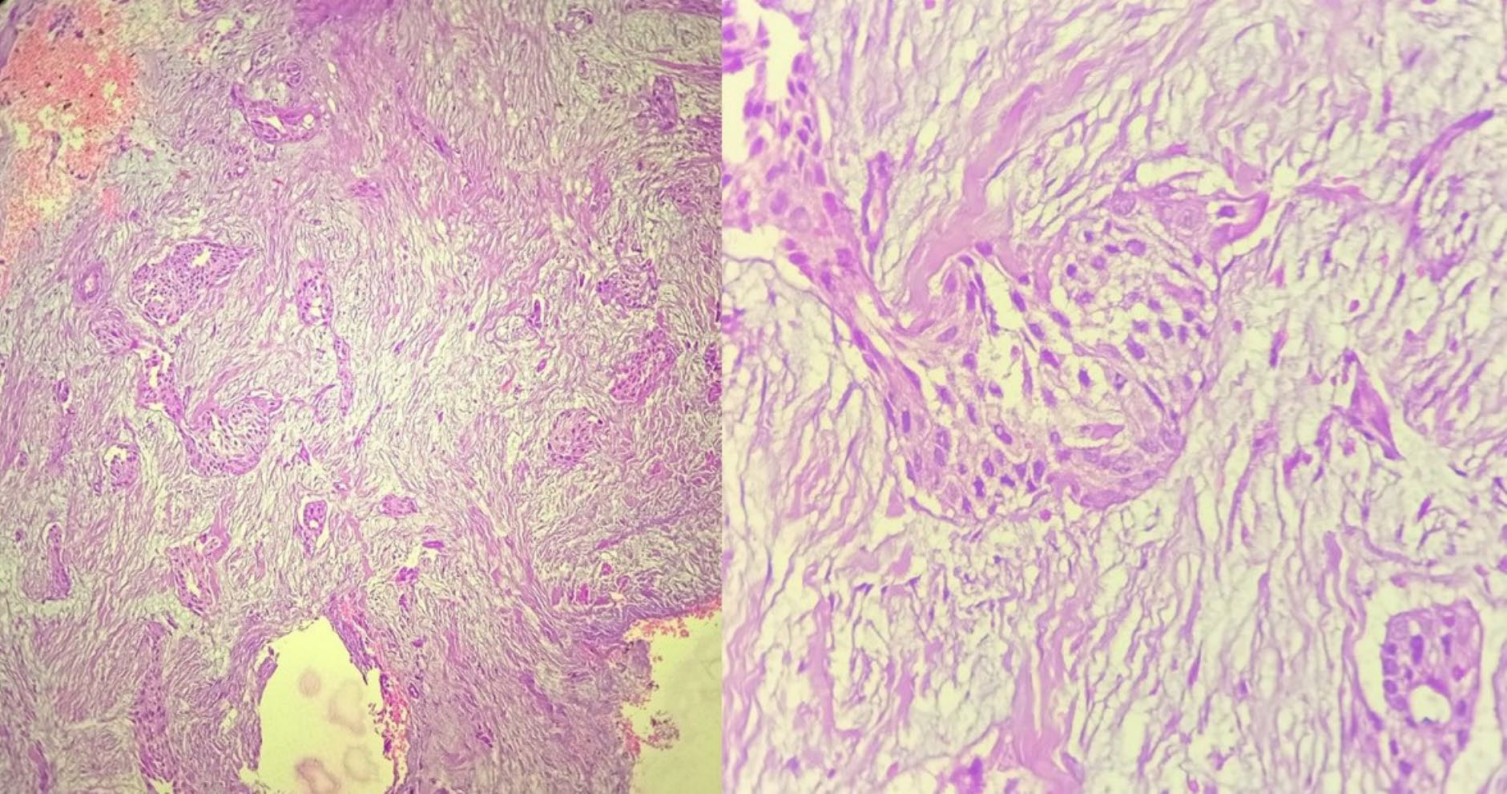
HPE showing fibromuscular tissue infiltrated by squamous cell carcinoma, moderately differentiated

Contrast-enhanced magnetic resonance imaging (CEMRI) of the orbit revealed a large irregular lesion (60 × 52 × 31 mm) in his left orbit predominately in the inferior part, involving inferior rectus muscle in its entirety up to orbital apex and extension infero-laterally through superior orbital fissure into left infra-temporal fossa, masticator space, left maxillary sinus with bony dehiscence in the posterior wall of left maxillary antrum, inferior aspect of lateral orbital wall. The left pterygoid muscle also involved. The cavernous sinus was spared and no intracranial extension was seen (Fig. 1). Biopsy confirmed squamous cell carcinoma of the orbital wall (Fig. 2). Following this, the patient was referred to us for further management.

**Fig. 3:**
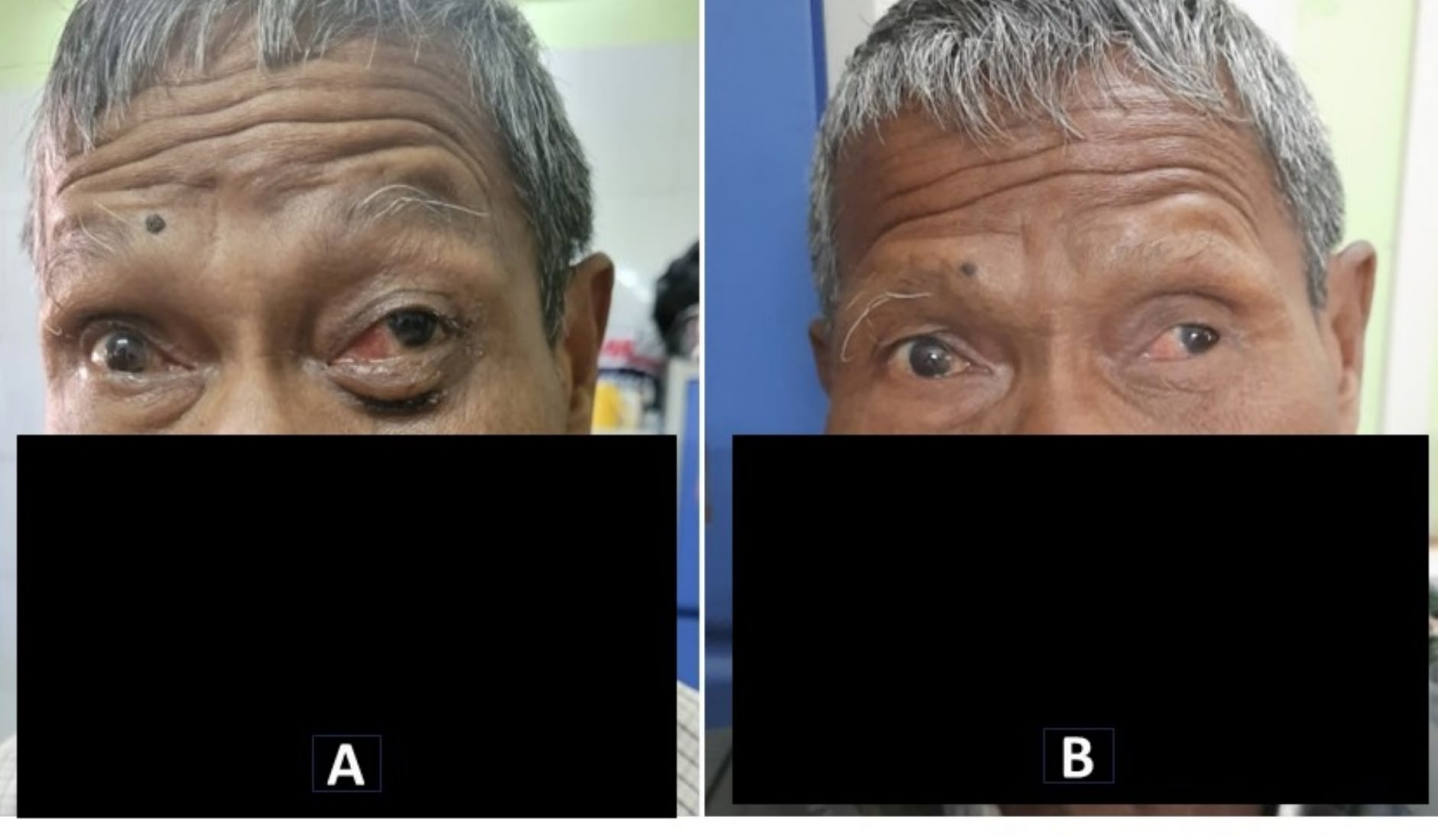
Clinical image of the patient: **a** pre-treatment image showing swelling and redness of the left eye; **b** 15 months post-treatment showing good clinical response

At that time, the patient also developed diffuse swelling over the right eye (Fig. 3A) and right side of the face along with the existing symptoms. PET-CT was done which showed 3.7 × 3.4 × 4.5 cm mass in the left retrobulbar region, left maxillary sinus extending to the infra-temporal fossa (ITF) (SUV max11.9) (Fig. 1). No evidence of distant metastasis was seen.

The case was then discussed in an institutional multidisciplinary tumour board. Surgical opinion was taken and he was deemed inoperable as R0 resection was found not feasible. He was then planned for definitive radiotherapy with concurrent chemotherapy.

**Fig. 4:**
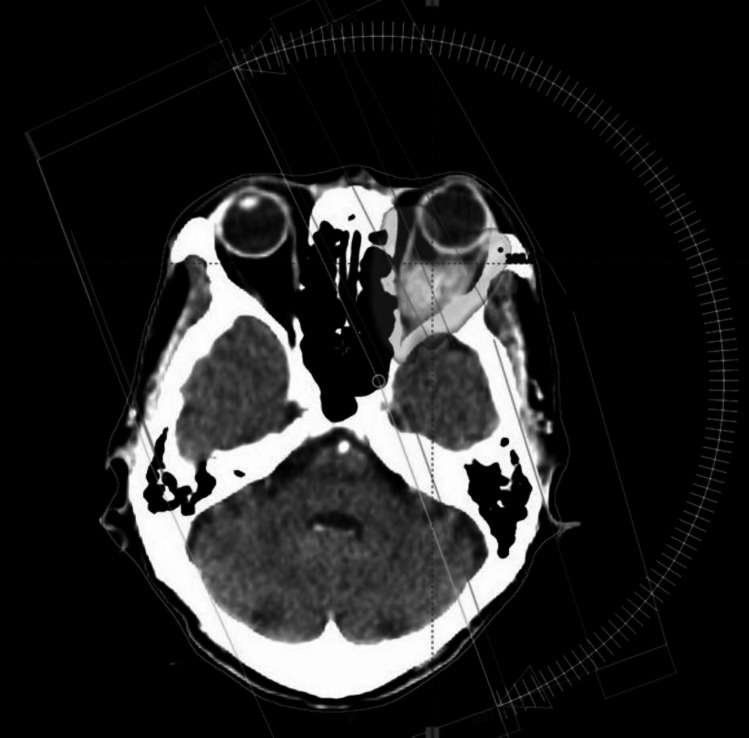
Axial dose wash of volumetric modulated arc radiotherapy treatment plan

Radiotherapy: The patient was treated with external beam radiotherapy (EBRT) to a dose of 60 Gy in 30 fractions from 5/1/23 to 25/2/23 using VMAT technique (Fig. 4).

The patient was immobilised in the supine position using three clamp thermoplastic brain mold. Intra-venous contrast-enhanced CT images were acquired after ensuring normal renal function (serum creatinine level and creatinine clearance) using the PhilipsCT simulator with 3-mm slice spacing. In digital imaging and communication (DICOM) format, these simulation images were exported to the Varian Eclipse treatment planning system (TPS). The patient’s MRI and PET-CT images were fused with the planning CT images. The gross tumour volume (GTVp) was delineated with aid of these images. A high-risk CTV was created to include areas of microscopic tumour involvement with a margin over the GTV by 10 mm, excluding the regions of air or bone without evidence of tumour invasion. The planning target volume (PTV) was created by adding a 5-mm geometric expansion over CTV. Organs at risk (OARs) were contoured as per institutional protocol.

Dose prescription was 60 Gy in 30 fractions to in PTV. The planning objectives for PTV were set such that 95% of PTV volume should receive at least 95% of the prescribed dose. The maximum dose (V107%) was restricted to less than 1% volume of PTV.

Normal tissue tolerance of radiation dose was respected as per the Quantitative Analyses of Normal Tissue Effects in the Clinic (QUANTEC) data. Dose-volume parameters for OARs are set as the maximum dose (Dmax) of the spinal cord (< 45 Gy), brainstem (< 54 Gy), optic chiasm (< 54 Gy), optic nerves (< 54 Gy), eyeball (< 45 Gy), and lens (< 10 Gy), and (Dmean) for parotids, cochlea (< 45 Gy), etc.

VMAT planning was done using the Eclipse TPS. VMAT plan was delivered with 6-MV X-ray beams with Varian Trilogy linear accelerator. Treatment setup verification was done with CT image guidance. The protocol of image guidance was daily for initial 3 days and then twice in a week. Radiotherapy was delivered five fractions in a week.

Chemotherapy: 6 cycles of weekly concurrent chemotherapy with 60 mg cisplatin @40 mg/m^2^ was delivered during RT.

Follow‐up and assessment: The patient was clinically assessed weekly during the treatment period. The patient tolerated the treatment well. At conclusion, the patient had acuity of vision within normal limits. After completion of treatment, the patient was subsequently followed up every 2 months by means of complete physical examination and imaging. The patient experienced occasional eye pain but maintained normal vision during follow-up visits.

**Fig. 5:**
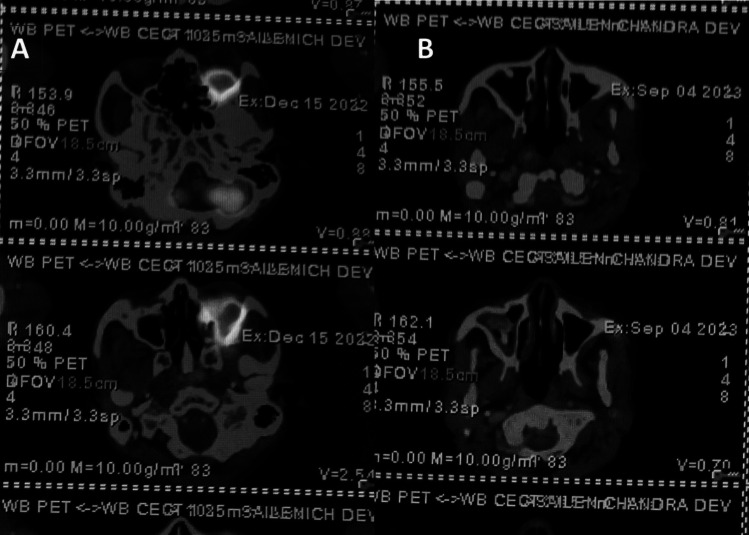
PET-CT images comparing pre-treatment and post-treatment. **a** Pre-treatment images showing the disease; **b** 6-month post RT showing complete resolution of the disease

At the first follow-up after 2 months of completion of RT, an MRI was done which showed residual lesion measuring 24 × 28 × 19 mm in size in the retrobulbar space of the left orbit in its inferior aspect involving both intraconal and extraconal regions along with post-treatment changes. The patient then came for second follow-up after 3 months; he had normal vision and no fresh complaints back then. A whole-body PET-CT scan was advised at 6 months of treatment completion. Compared to previous scans, the PET-CT scan showed a complete metabolic response (Fig. 5).

The patient continued routine follow-up appointments, with complete clinical and radiological response till last follow-up which was 15 months post radiotherapy (Fig. 3B). Ophthalmology consultation was advised for cataract screening and power adjustment, with regular follow-ups scheduled in the radiation oncology department.

## Discussion

Primary squamous cell carcinoma (SCC) originating in the orbit is an exceedingly rare entity, with only less than 20 documented cases in the medical literature. This scarcity is likely attributable to the absence of native squamous epithelium in the orbit. Secondary orbital SCC can be attributed to local invasion of SCC of the skin, paranasal sinus, nasopharynx, nasal cavity, conjunctiva, lacrimal glands or sacs, or occult extension by perineural spread; or hematogenous metastasis. Metastatic tumours are usually poorly differentiated and carry a poor prognosis with patients surviving only a few months or a year [2–7].

The pathogenesis of primary orbital SCCs is hypothesised to arise from malignant transformation of dermoid cysts, squamous metaplasia of lacrimal gland cysts, or following malignant transformation of iatrogenic transplanted conjunctival epithelium into the orbit during ocular surgical procedures [8, 9].

A comprehensive review of previous cases in the literature reveals substantial heterogeneity in demographic, clinical, and therapeutic profiles among reported cases. Variability is noted in patient age (ranging from 43 to 99 years), presenting symptoms (including pain, numbness, proptosis, ptosis, diplopia, and visual disturbances), tumour location (apical and non-apical), and treatment modalities (such as simple debulking, orbital exenteration, craniofacial resections, adjuvant chemotherapy or RT, or primary chemoradiotherapy) as outlined in Table 1. Consequently, a defined age group, characteristic symptomatology, or standard treatment approach cannot be universally established.

**Table 1:** Clinical and treatment details of published cases of primary squamous cell carcinoma of the orbit

Sl No	Author/year	Age/sex	Presenting complaint	Treatment details	Follow-up	Status at FU
1	Peckinpaugh JL et al. (2012) [2]	43/F	Diplopia, ptosis	Definitive RT > adjuvant CT	49	Alive NED
2		63/M	Diplopia	Definitive RT > adjuvant CT	19	Died
3		67/M	Diplopia, ptosis	Definitive RT only	12	Died
4	Ruff T et al. (1985) [3]	53/F	Facial pain	Excision > adjuvant RT	84	Alive NED
5	Saha K et al. (2011) [4]	56/F	Facial numbness, diplopia, ocular pain	Excision > adjuvant RT	NR	NR
6	Campos Arbulú AL et al. (2017) [5]	73/F	Eye pain, headache	Orbital exenteration > adjuvant RT	34	Alive NED
7	Blandford AD et al. (2018) [6]	63/M	Pros is, facial numbness, diplopia	Excision > adjuvant RT + CT	16	Alive NED
8	Choi DY et al. (2014) [7]	74/F	Diplopia, eye pain, facial numbness	Orbital exenteration > adjuvant RT	17	Alive NED
9	Krishnamurthy S. et al. (2023) [10]	65/F	Orbital pain, loss of vision, red eye	Definitive RT only	24	Alive NED
10	Hromas AR et al. (2014) [11]	43/M	Decreased vision, ophthalmoplegia	Orbital exenteration > adjuvant RT	24	Alive NED
11	El Samkary M et al. (2022) [12]	99/F	Proptosis and facial numbness	Orbital exenteration	6	Alive NED
12	Karrabi N et al. (2022) [13]	45/F	Orbital pain, periorbital fullness	Tumour debulking > adjuvant RT > adjuvant CT	2	Alive PD
13	Lim YJ et al. [14]	F	Proptosis, blurring of vision	Only palliative care	1	Died
14	Kennedy A et al. [15]	78/M	Proptosis, ptosis	Palliative surgery	-	Died PO day 2
15	Md. Rahman et al. [16]	62/M	Swelling, proptosis	Definitive RT + concurrent CT > salvage surgery at 3 months	32	Alive NED
16	Present case	60/M	Blurred vision, redness in the eye, headache	Definitive RT + concurrent CT	15	Alive NED

*M* male, *F* female, *NED* no evidence of disease, *PD* persistent disease, *RT* radiotherapy, *CT* chemotherapy, *FU* follow-up, *NR* not recorded

Of the described 16 cases including the current case, nine patients underwent surgical intervention with or without adjuvant therapy. Notably, six patients were treated with upfront definitive RT with or without concurrent and adjuvant chemotherapy. Out of these six patients treated with upfront RT, four patients were alive without any evidence of disease at their last recorded follow-ups. Typically, radiation doses for orbital tumours of histologies such as retinoblastoma, lymphoma, meningioma, and rhabdomyosarcoma are limited to below 40–50 Gy owing to their radio-sensitivity and associated risk of ocular toxicity [10]. Many a time, the intent of radiation is also palliative rather than curative. In the current case, the decision to escalate the total dose to 60 Gy at 2 Gy per fraction was influenced by the use of RT as the definitive treatment modality and the SCC histology, aligning with standard curative doses for head-and-neck SCC. Concerns regarding potential severe ocular toxicity, such as panophthalmitis, were carefully considered given the risk of exacerbating existing symptoms [11–16].

## Conclusion

Primary SCC of the orbit is a rare entity with only a handful of cases reported till date. As evident from the systemic review of literatures, the treatment options are varied. Radiotherapy can successfully treat such cases with acceptable treatment-related toxicity and preservation of vision.
